# Traditional and complementary medicine use among Ebola survivors in Sierra Leone: a qualitative exploratory study of the perspectives of healthcare workers providing care to Ebola survivors

**DOI:** 10.1186/s12906-020-02931-6

**Published:** 2020-05-06

**Authors:** Peter Bai James, Jon Wardle, Amie Steel, Jon Adams, Abdulai Jawo Bah, Stephen Sevalie

**Affiliations:** 1 Australian Research Centre in Complementary and Integrative Medicine, School of Public Health, Faculty of Health, University of Technology Sydney, Ultimo, Sydney, NSW 2007 Australia; 2 Faculty of Pharmaceutical Sciences, College of Medicine and Allied Health Sciences, University of Sierra Leone, Connaught Teaching Hospital Freetown, First floor Administrative Building, Freetown, Sierra Leone; 3 National Centre for Naturopathic Medicine, Southern Cross University, Lismore, NSW 2480 Australia; 4 Faculty of Basic Medical Sciences, College of Medicine and Allied Health Sciences, University of Sierra Leone, Freetown, Sierra Leone; 5 Institute for Global Health and Development, Queen Margaret University Edinburg, Musselburgh, Scotland, UK; 6 Joint Medical Unit, Republic of Sierra Leone Armed Forces, 34 Military Hospital Wilberforce Freetown, Freetown, Sierra Leone; 7 Sustainable Health Systems, Freetown, Sierra Leone

**Keywords:** Ebola, Ebola survivors, Health personnel, Traditional medicine, Complementary therapies, Perception, Attitude, Communication, Integration, Sierra Leone

## Abstract

**Background:**

Considerable number of patients, including Ebola survivors, in Sierra Leone, are using traditional and complementary medicine (T&CM). Healthcare providers’ (HCPs) views about T&CM is crucial in addressing the increased need for T&CM among patients. However, healthcare providers’ views about T&CM in Sierra Leone is unknown. Our study explores healthcare providers’ knowledge of and perception towards T&CM and how that influence their personal and professional T&CM use, communication with Ebola survivors about T&CM as well as its integration into the healthcare system in Sierra Leone.

**Methods:**

We employed a qualitative exploratory study design using semi-structured interviews to collect data from 15 conveniently sampled HCPs in all four geographical regions of Sierra Leone. We analysed our data using thematic network analysis framework.

**Results:**

Healthcare providers perceived their knowledge about T&CM to be low and considered T&CM to be less effective and less safe than conventional medicine as well as not evidence-based. HCPs perception of T&CM as non-scientific and their lack of knowledge of T&CM were the key barriers to HCPs’ self-use and recommendation as well as their lack of detailed discussion about T&CM with Ebola survivors. HCPs are open to T&CM integration into mainstream healthcare in Sierra Leone although at their terms. However, they believe that T&CM integration could be enhanced by effective professional regulation of T&CM practice, and by improving T&CM evidenced-based knowledge through education, training and research.

**Conclusion:**

Changing HCPs’ negative perception of and increasing their knowledge about T&CM is critical to promoting effective communication with Ebola survivors regarding T&CM and its integration into the healthcare system in Sierra Leone**.** Strategies such as educational interventions for HCPs, conducting rigorous T&CM research, proper education and training of T&CM practitioners and effective professional regulation of T&CM practice could help in that direction.

## Background

The growth of the traditional and complementary medicine (T&CM) industry in the past two decades has attracted the attention of healthcare providers, researchers, policymakers and consumers around the world [1, 2]. In Africa, people used T&CM predominantly to address their primary healthcare needs. For instance, a recent systematic review suggests that more than half of the population used T&CM [3]. The key drivers of T&CM use in Africa include availability, accessibility, its perceived safety and efficacy, link with cultural and spiritual values, and patient despair with conventional medicine [3]. Sierra Leonean studies have reported high use of T&CM among pregnant women [4], lactating mothers [5], hypertensive patients [6], healthcare students [7, 8], infertile women [9] and among children and adults with malaria [10, 11].

Compared to the current and previous Ebola outbreaks in East Africa, the 2014–2016 Ebola epidemic in West Africa recorded not only the highest number of cases and deaths but also the largest number of survivors [12–14]. The number of Ebola survivors in West Africa is estimated to be more than 10,000 [15]. As one of the hardest hit countries in West Africa, Sierra Leone is host to approximately 4000 Ebola survivors [16]. Research has reported that the majority of Ebola survivors are experiencing a lot of physical and psychosocial health challenges, some of which are severe and chronic [17, 18]. Ebola survivors have been reported to be practising medical pluralism (use of conventional and indigenous healthcare systems) to address their health challenges during and after the Ebola outbreak in Sierra Leone. A recent Sierra Leonean study suggests that close to half of Ebola survivors used T&CM to manage their post-Ebola sequelae [19]. Herbal medicine was the most common T&CM modality employed by Ebola survivors [19]. Joint pain and abdominal pain were the most common post-Ebola indications for T&CM use [19]. Ebola survivors were attracted to using T&CM because of personal beliefs, economic, psychological, social and cultural needs as well as health system factors [19–22]. These factors include the ineffectiveness of conventional medicine, healthcare providers’ negative attitudes toward Ebola survivors, high cost of, and unavailability of conventional medicine to manage post-Ebola symptoms and T&CM being in line with Ebola survivors’ culture and tradition [19, 21, 22]. Also, Ebola survivors’ beliefs regarding T&CM have reported influencing their decision to use T&CM. These include the view that T&CM helps boost survivors’ immune systems, T&CM being natural and relatively safe than conventional medicine, T&CM provides survivors with more control over their health as well as the relative importance of personal experience of T&CM effectiveness based on prior use when compared to clinical evidence [20].

The use of T&CM among Ebola survivors may present direct and indirect risks that could undermine their health outcomes and quality of life [23, 24]. Direct risk includes inherent adverse effect, toxicity or adverse effects due to conventional-T&CM drug interactions and heavy metal contamination [23]. Indirect risk includes lack of education and training of T&CM practitioners as well as poor T&CM regulation [23–25]. The risk that T&CM posed to Ebola survivor’s safety may be exacerbated, given that the majority of patients in Sierra Leone do not disclose their use of T&CM to healthcare providers (HCPs). HCPs need to proactively initiate discussions with survivors about T&CM and be in a position to appropriately advice on the risks and benefits associated with T&CM use in order to protect Ebola survivor’s safety and maximise their health. Such T&CM competency requires HCPs to be knowledgeable about commonly used T&CM. However, studies have shown that HCPs are less knowledgeable about T&CM, which limit their ability to provide appropriate advice to their patient regarding T&CM, use [26–30].

Lack of knowledge and negative perception of T&CM among HCPs are known to limit T&CM integration into the healthcare system [28, 29]. HCPs are known to stigmatise T&CM practices and practitioners by tagging them with names such as “unconventional”, “unproven”, “pseudoscience” and “quackery” all of which undermine efforts to integrate T&CM into the mainstream healthcare system [31, 32]. In Sierra Leone, biomedical and T&CM health systems exist parallel to each other with biomedicine being the recognised and dominant healthcare system. In line with WHO recommendations, a national traditional medicine policy was designed, which recognises the need for T&CM integration into the healthcare system [33]. However, no attempt has been made so far to implement such a policy.

Currently, it is unclear how HCPs communicate with patients, including Ebola survivors about T&CM and whether HCPs favour T&CM integration into the healthcare system in Sierra Leone. HCPs’ knowledge and perception of T&CM will largely influence their nature of communication with patients about T&CM and its integration into the healthcare system in Sierra Leone.

Previous Sierra Leonean studies [19–22] have established that Ebola survivors practice medical pluralism in addressing their healthcare needs, which has the potential to undermine their safety. Personal and health system factors, including the negative attitudes of healthcare providers towards survivors, were cited as reasons why Ebola survivors decided to seek T&CM. These Sierra Leonean studies also found that disclosure of T&CM use among Ebola survivors was low, which is an indication that communication about T&CM use between healthcare providers and Ebola survivors is not optimal [19–22]. With these issues and circumstances in mind, we wanted to understand in this study how healthcare providers interact with Ebola survivors regarding T&CM. Thus, the objective of our study was to explore HCPs’ knowledge and perception towards T&CM and how that influence their personal and professional T&CM use, communication with Ebola survivors about T&CM as well as its integration into the healthcare system in Sierra Leone.

## Methods

### Study design and setting

We used a qualitative content analysis exploratory study design using a semi-structured interview guide to collect our data. The study was conducted in four districts covering the four geographical regions of Sierra Leone, namely Freetown in Western Area, Bo district in the southern region, Kenema district in the eastern region and Bombali district in the northern region.

### Participants and sampling procedure

Healthcare workers providing care to Ebola survivors were invited to participate in our study through snowball sampling. Recruitment of HCPs to participate in the study was done via telephone. The HCPs in our study included doctors, nurses and community health officers. The final sample (*n* = 15) was established when we reached data saturation during data collection.

### Data collection procedure

The first author (PBJ) used a pretested semi-structured interview guide to collect information from the study participants between May and August 2018. The first author is a Sierra Leonean male with a pharmacy and public health background. He is studying for a PhD in public health at the time of conducting the study. He is trained in qualitative research and had experience in conducting semi-structured interviews among health professionals and patients in Sierra Leone. We developed the interview guide based on the available scholarship on HCPs knowledge, attitude and practice of T&CM as well as their views regarding its integration with conventional medicine [26–30, 34–38]. The guide was pretested among three HCPs, and their response helped to inform the design of the final version of the interview guide. Their responses were excluded in the final data analysis. The interview guide consisted of open-ended questions that explored the HCPs’ knowledge, perceptions and beliefs about T&CM as well as their personal and professional use of T&CM. Issues surrounding HCPs’ communication with Ebola survivors regarding T&CM and their perspective on the integration of T&CM in the national healthcare system in Sierra Leone were examined. We defined T&CM in our study to include herbal medicine, animal extract, traditional medicine practice (traditional bone setting and scarification and local surgery), prayer/faith healing and massage.

The first author conducted face-to-face semi-structured interviews in English. The interviews were conducted in their offices or consulting room after working hours, and it lasted between 30 and 95 min and were digitally recorded. In line with good qualitative interview fieldwork, the interviews provided a full opportunity for interviewees to introduce issues/areas that they perceived as central/significant to the topic. As such, the duration of each interview was varied, and in some cases, the length of the interview was relatively extensive (with the interviewee’s permission/consent). We took field notes during and immediately after each interview to help confirm the accuracy of the interview transcripts and to aid data interpretation. The contents of the field notes were included in the final data analysis. The first author transcribed the digital recordings verbatim in English. The transcripts were sent via email to all participants to get their feedback regarding the accuracy of their response to the issues discussed during the interview. However, only six were able to respond. We did not conduct any repeat interviews, given that all the relevant areas were discussed in all of the 15 interview sessions.

### Ethical considerations

The University of Technology Sydney Human Research Ethics Committee (UTS-HREC-ETH17–2080) and the Sierra Leone Ethics and Scientific Review Committee provided the approval for the conduct of the study. Before the start of the interview, HCPs were given a participants information sheet that explains the nature and scope of the study as well as the researchers’ interest in the research topic. The participant’s information sheet also makes it clear that participation is voluntary, and that they had the freedom to withdraw from the study without any reason. After reading through the participant information sheet and verbally agreed to participate, HCPs were given a consent form to sign. Signing the consent form was interpreted as a confirmation of HCPs’ willingness to participate in the study.

### Data analysis

A framework method using an inductive approach [39, 40] was used to analyse our data. We used N-Vivo Version 11 software to manage and analyse our data. The authors read all the transcripts, and familiarise themselves with issues discussed during the interviews as well did the initial coding. The first author developed the framework, indexing, chatting, mapping and interpretation of the data while the second, third and fourth authors supervised the coding, categorisation and theme identification processes. In instances were varied viewpoints existed, they were addressed through discussion, and a consensus was reached among the four authors.

## Results

Table 1 gives the socio-demographic characteristics of conventional healthcare providers that were interviewed. The key themes that emerged from the analysis of our data include perception of, and knowledge about T&CM, experience of personal use and recommendation, reasons why Ebola survivors use T&CM, communication with Ebola survivors about T&CM use and T&CM integration within the Sierra Leone healthcare system. This study adheres to the Consolidated Criteria for Reporting Qualitative Research (COREQ). Please see supplementary file 1 for details. We used representative quotes to illustrate the views of healthcare providers. We also added pseudonyms to these quotes to ensure participant anonymity.

**Table 1:** Demographic Characteristics of Healthcare Providers (*n* = 15)

Characteristics	Variables	n (%)
Age group (years)	20–29	3 (20.0)
	30–39	6 (40.0)
	40–49	4 (26.7)
	50+	2 (13.3)
Sex	Male	8 (53.3)
	Female	7 (46.7)
Length of experience (years)	0–5	7 (46.7)
	6–10	6 (40.0)
	11+	2 (13.3)
Cadre	Doctor	5 (33.3)
	Nurse	5 (33.3)
	Community health officer	5 (33.3)
Geographical Region	Western Area	5 (33.3)
	Southern province (Bo District)	3 (20.0)
	Eastern province (Kenema district)	4 (26.7)
	Northern province (Bombali District)	3 (20.0)
Practice setting	Hospital	10 (66.7)
	Community	5 (33.3)

### Perception of and knowledge about T&CM

HCPs generally considered T&CM to be less effective, less safe, and without any dose or strength compared with conventional medicine and therefore, can lead to serious adverse health outcomes. Some believe that traditional medicine is just a money-making venture. Many believed that T&CM use is one of the major causes of Ebola survivors' delay in seeking conventional healthcare. As such, HCPs always advise their patients not to use T&CM but first to seek care at the hospital when they are sick. Healthcare providers also mentioned that the piece of advice they gave to their patients not use T&CM was based on personal experience with patients who have used T&CM and that research has not proven that T&CM is more effective than conventional medicine. They also cited that the use of T&CM promotes non-adherence to conventional therapy.

*“As far as I am concern, TM is not good for once health. I have seen where traditional medicine has caused problems for patients. For me, traditional medicine is more of guesswork. Most of the time, T&CM practitioners are not sure whether traditional medicine will work or not. For western medicine –if they say this paracetamol cures headache and you take it, after 5, 10, 15 minutes, you will feel ok, and you see its effect. Whereas for TM, you are not sure whether that will happen and may even cause liver damage.” [P9]*

HCP asserted that their knowledge about T&CM is limited. They attributed their lack of knowledge about T&CM to the subject not being taught during their training at the university and lack of research on T&CM in Sierra Leone. HCPs were told that they should advise patients not to take T&CM as it could adversely affect the health of the patient. However, HCPs mentioned that they were willing to know more about T&CM and open to recommending the use of T&CM if evidence of its safety and effectiveness is made available*.*

*“No, we were not taught anything about T&CM. We never had any module on that (T&CM). What we were told was that we should always advise people not to use T&CM”. Even after I graduated, I have not heard or read about any study on T&CM in Sierra Leone that has shown to be more effective or safer than conventional medicine. I will like to learn more about the safety and efficacy of the frequently used T&CM so that I can advise patients accordingly. [P4]*

### Experience of personal use and recommendation

All of the HCPs mentioned that generally they do not personally use, or recommend the use of T&CM to survivors. HCPs perception of T&CM as non-scientific and their lack of knowledge of T&CM were the key barriers to HCPs self-use and recommendation for use to survivors. Some, however, mentioned that they have used and recommended certain types of T&CM modalities such as prayer/spirituality, massage therapy, as they considered them useful and not harmful.

*“As for me, I do not believe it [traditional medicine] works because it has not been scientifically proven, and I have no knowledge of what it is composed of. So, I do not take it [traditional medicine] or recommend it, my patients.”[P1]*

*“Well, I am a religious person so, I sometimes fast and pray to receive divine healing from God when I am sick, and I often recommend it to my patients in addition to the treatment I give to them. In addition to the medications I prescribe, I sometimes recommend massage therapy to survivors who present with back pain or muscle pain. As far as I am concerned, these are OK for use because they do not cause any problem and they do work” [P7]*

### Reasons why survivors use T&CM

HCPs cited affordability, availability, tradition, lack of awareness about the safety of T&CM, family or peer influence and survivors’ perceived stigma at the hospital as the key reasons why Ebola survivors decide to use T&CM*.*

*“Well, lack of money to pay hospital bills such as drugs and laboratory services. Also, traditional beliefs is another factor. Sometimes, they have the money, but if they believe that this particular disease is best cured if I use traditional medicine, he/she will use it”.[P13]*

*“I did remember we had a couple of them who decided not to come to clinic but instead use T&CM because they thought they would be stigmatised or discriminated or refused to be treated if they come to the clinic. [P1]*

### Communication with Ebola survivors about T&CM use

Majority of HCPs described that, as part of their medication history taking, they routinely ask survivors about their T&CM use status knowing that patients commonly use T&CM before visiting the hospital*.*

*“That is part of our history taking. When we see them (Ebola survivors), we ask to know what type of drugs they have used, and that includes native herbs as well because we are aware that it is a common practice” [P5]*

Only one confessed that he does not usually ask about survivor T&CM use

*“I usually do not ask about that. It is not an issue that I discuss with them. It is good you have reminded me about that. I will be asking now” [P6]*

As to whether they felt comfortable discussing in detail about T&CM with Ebola survivors, the majority of them mentioned that they do not feel comfortable discussing T&CM as they lack evidenced-based knowledge. Competing demands for time was also a barrier to the extent to which HCPs can discuss with Ebola survivors about T&CM. For instance, HCPs mentioned that they do not see the need to spend time discussing T&CM further with Ebola survivors knowing that they would eventually advise Ebola survivors against using T&CM and that such time could be used to see other patients.

*“ … If they are taking western medicine, we can discuss further the benefits and risks of taking it and advice accordingly because I know a lot about western medicine. But with T&CM, that is not my area of expertise. All that I do is to know whether they [Ebola survivors] have taken traditional medicine. If yes, I then asked about the name and quantity consumed and where they got it. After that, I advised them to stop taking it because generally, we know traditional medicine is not good for their health. I have witnessed several times where it [traditional medicine] has caused problems such as damage to the liver” [P8]*

As to whether survivors are truthful about their use of T&CM, some HCPs mentioned that survivors do not hesitate to disclose the TM use status. On the other hand, some HCPs believed that some Ebola survivors were not willing to disclose their TM use even when asked.

*“If they have used it, they will tell us. It is not something they will refuse to talk about. They will say –for example –when I had a headache; I took so and so TM. They will even say and I quote “ na kontri meresin na e ar be dae use but wae ar nor see betteh na im make ar cam na hospital ”(I was using TM before coming to the hospital but because I did not observe any improvement in my condition made me come to the hospital)” [P5].*

*“But as you know the culture of our people-not everybody will be honest enough to tell you if they (Ebola survivors) take traditional medicine more, especially if they come in that acute condition. They will hardly tell you if they have taken traditional medicine” [P15]*

### HCPs reaction to Ebola survivor’s use of T&CM

HCPs stated that their reaction to survivor’s use of T&CM is one of acceptance that the deed has been done and trying to understand the reason(s) for use, the type and amount used as well as advising them to stop using it, and first to seek care at the hospital whenever they are sick.

*“For a clinician, you accept it. You do not want to disown them and say well go back to your traditional medicine –you encourage them and tell them that next time come to the clinic when you are sick other than taking herbal drugs”* [*P1]*

HCPs stated that their manner of communication in convincing survivors to stop using T&CM is one that is full of empathy and respect*.*

*“I do not yell or shout at them. I usually talk to them [Ebola survivors] politely because I am trying to convince the patient to believe what I am telling him/her. It is his/her belief that T&CM is good, and it works. Therefore, you have to talk to him/her politely to change his/her behaviour, convince him, and show him the reasons why he needs to stop taking TM. If you explain to him and he understands and agrees with you, he would follow your advice. But if you yell at him, he/she will not follow your advice” [P7]*

### T&CM integration within the healthcare system

Majority of HCPs interviewed recognised the need for collaboration with T&CM practitioners given that T&CM use and practice is widespread. HCPs are aware that T&CM practitioners are close to the community and therefore, serve as the first port of call when people are sick. Healthcare providers mentioned that T&CM practitioners’ role in stopping the spread of the Ebola virus further highlighted the need for conventional medicine to collaborate with T&CM

*“Yeah, I believe they have a role to play because they are in the community, they live with the people and interact with them on a daily basis. So when someone from such a community gets sick, the first point of contact is that traditional medicine healer that stays is close.” [P3]*

*T&CM is an essential aspect of healthcare in this country. Many people practice it. To say not to collaborate with the T&CM system means to allow the status quo to continue and which is not favourable at all. As a country, we started making the right moves years back when a national Traditional Healers association was formed, and we realise the need of this association during the Ebola crises. Initially, the chain of Ebola transmission was through T&CM practitioners. It was not until T&CM practitioners came on board and decided to put a hold on their practice, that we started seeing cases coming to our facilities and getting the appropriate treatment. If we did not have such an association beforehand, it would have been very difficult for us to get the cooperation from each T&CM practitioners in the country. That has taught us a good lesson that we cannot ignore them”. [P2]*

However, HCPs’ demonstrated varied understanding of what collaboration or integration should look like between healthcare providers and T&CM practitioners. For instance, the majority cited that collaboration should take the form in which T&CM practitioners should refer patients to HCPs who present with a disease that is beyond their expertise but not the other way round.

*“I believe that they have to play more of a referral role because if they have any case that is beyond their expertise, they need to refer to us here or bigger hospital for further treatment.” [P8].*

Only one believed that collaboration should involve the sharing of ideas and that patient referral should occur both ways

*“For me, I think it is good for traditional medicine to be associated with western medicine. If there are close to us, we can share ideas. When they realise that certain cases are above them, they refer the patient to us and when we also have done all that is necessary for a patient, but he/she is not improving, we will refer the patient to THP to assess the patient.”[P7].*

Some HCPs cited that integration should be allowed for mind-body T&CM therapies such as prayer/spiritual therapies as it is considered safe. However, collaboration should not be allowed for T&CM approaches such as herbal medicine that is perceived to cause serious adverse effects.

*“Some patients will say they want to go to church for prayers. We do allow them to go to church or sometimes, the pastor will come to the hospital and pray for them. The thing is that prayer affects the mind, not the physical body, as is the case of herbal medicine. For me, prayer is one way to counsel someone; it is a way to reduce stress. Therefore, such collaboration between us [Healthcare providers] religious leaders with regard to the treatment of a patient and us is ok for me.”[P9]*

Others also believed that integration requires the training of T&CM practitioners on what diseases to treat and those that require referral. It also requires the regulation of T&CM and public education on the need to seek care to only licensed T&CM practitioners*.*

*I was reading one model of integration in Tanzania. In mountainous areas where no healthcare worker is ready to go, but they have T&CM practitioners who have been working there for years. These T&CM practitioners were trained on what to treat and when to refer and were given certificates at the end of the training. Also, a whole lot of sensitisation went around that if you are sick and you want to visit a traditional healer, make sure he/she is licensed, as these are the ones that have been trained and at least given some basic medical training including when to refer. Therefore, I think it is something we need to look into and try to get other models that have been working in other countries; we study them and see how it can be adapted. [P2]*

## Discussion

This study is among the first in Africa to explore the views of healthcare providers on T&CM use among Ebola survivors. HCPs’ negative perception of T&CM in our study is in line with similar studies among health subpopulations in Africa [28–30, 35] and it underscores the scepticism among the biomedical community regarding the safety and efficacy of T&CM therapies. This scepticism in our study is based on HCPs firsthand experience of the ineffectiveness and adverse effects of T&CM use among their patients and the general lack of scientific basis for T&CM use. HCPs advice to patients to seek care at the hospital instead of using T&CM reflects their perceived superiority of biomedicine over T&CM. In Sierra Leone, biomedical care is associated with modernity, technology and science and therefore credible whereas, T&CM is considered non-scientific, quackery and lacks legitimacy as a credible healthcare option. For healthcare professionals, this association to science and technology is essential and may influence their perception of, and recommendation for the use of T&CM. The direct (adverse effect, toxicity and heavy metal contamination), indirect (models of disease causality and treatment, lack of standardised education and training and poor regulation) risks to survivors’ safety that T&CM is known to possess may further explain HCPs’ negative perception of T&CM in our study [23, 24].

Numerous studies have reported the limited knowledge of T&CM among healthcare providers [29, 30, 35], and our study is no exception. The perceived non-scientific nature of T&CM may explain why it was not part of HCPs training in our study. However, given the high use of T&CM in the society and the potential adverse health outcome among patients when conventional medicine and T&CM are used concurrently, underscore the need for HCPs to be knowledgeable about the commonly used T&CM by their patients including Ebola survivors. HCPs’ willingness to know more about T&CM reflects the need for T&CM to be included in the curricula of healthcare training institutions in Sierra Leone. Similar demands for evidence based T&CM training in healthcare training institutions have been made by healthcare students in Sierra Leone [7, 8]. Continuing education programs, training workshops and the development of reputable pharmacopoeias for referencing at public health facilities are strategies that can be used to improve HCPs’ knowledge about commonly used T&CM. Also, the development of evidence-based factsheets on T&CM approaches can help improve HCPs’ knowledge and HCP- patient communication about T&CM.

Previous research has shown that HCPs with increased T&CM knowledge are more likely to engage in conversations regarding T&CM with their patients [41]. The need for evidence based T&CM knowledge among HCPs in our study also requires that rigorous research need to be conducted on the safety and efficacy of commonly used T&CM therapies in Sierra Leone. Findings from such research will inform policy formulation with regards T&CM use in mainstream healthcare. However, as in other countries, interest in and funding for T&CM research still lags behind that for conventional medicine [42, 43]. Going forward, funding for preclinical, clinical and health service research are warranted in order to develop evidence based T&CM scholarship, which could inform T&CM integration- where possible into mainstream healthcare.

HCPs views as to the reasons why Ebola survivors used T&CM in our study are in line with common drivers of T&CM in the general and sub-health populations in sub-Saharan Africa [3] and among Ebola survivors specifically [19, 21, 22]. Our finding also shows that HCPs recognised that their negative attitude towards survivors is a potential driver for T&CM use among Ebola survivors. HCPs negative attitude can lead to patient dissatisfaction with conventional healthcare, and that has been identified as a driver for T&CM use among Ebola survivors [21, 22]. It is reported that Ebola survivors experience widespread stigma and discrimination from the community and hospital staff following their discharge from the Ebola treatment centre [17, 22]. Education interventions to change HCPs’ negative attitude are required. In addition, anti-stigmatisation and discriminatory laws need to be created and implemented that create a save space for Ebola survivors to receive care without being stigmatised or discriminated.

Effective communication about T&CM with Ebola survivors is critical to delivering safe care and enabling Ebola survivors to make informed therapeutic decisions [44]. Consistent with previous findings [25, 34, 45], our qualitative data indicates that despite the majority of HCPs said they routinely initiate T&CM discussion with Ebola survivors during consultations, majority of HCPs do not feel comfortable discussing in details about T&CM that Ebola survivors are using due to lack of knowledge. In line with an Australian study among oncologists [46], HCPs discussion about T&CM use with Ebola survivors in our study was framed only in the context of risk given their general perception that T&CM use can lead to adverse health outcomes. Even though this might be true, HCPs should not dismiss all T&CM as less safe but actively seek to obtain information that could be useful to their patients in general and Ebola survivors in particular. HCPs dismissal of the use of a particular type of T&CM, especially ones that survivors believes is efficacious and safe could undermine effective communication due to mistrust and lead to Ebola survivor non-adherence to conventional therapy. HCPs should endeavour to initiate and maintain respectful empathic conversations that is devoid of prejudice and also sensitive to Ebola survivors’ past experience with T&CM.

As reported in previous research [25, 45], time constraint was also an impediment to effective communication regarding T&CM in our study. Previous studies have reported that professional and organisational governance affect HCP attitude towards T&CM [45, 47]. Therefore, strategies needed to promote communication regarding T&CM in the clinical setting should also factor HCP working environment.

Concurrent with previous studies [25, 45, 48], HCPs in our study described their openness to discuss T&CM with Ebola survivors following their disclosure of T&CM use. Such a positive reaction by HCPs will help enhance patient-healthcare provider relationship and patient satisfaction [48]. However, such positive response may have limited effect on patient health outcomes in the current healthcare environment, given that HCPs’ negative perception of, and limited knowledge about T&CM were identified as key barriers to effective communication in our study. This suggests that even with HCPs positive response to Ebola survivors’ T&CM disclosure, HCPs’ lack of knowledge and negative perception will undermine effective communication regarding T&CM. When HCPs are confronted with the task of providing information about a phenomenon that they have little or no knowledge of, they may feel incompetent. Such feeling can lead to HCP being defensive and, as such, undermines effective communication between HCPs and their patients [44, 49]. Therefore, effective communication regarding T&CM in clinical setting needs to be viewed beyond HCPs positive response to patient T&CM disclosure but should include a dialogue that is patient-centred, open and evidence-based [50].

In line with previous studies in Africa [28, 37, 38], HCPs acknowledged the need for collaboration with T&CM practitioners. However, they prefer the collaboration to be limited to one way referrals from T&CM practitioners to HCPs. HCPs’ scepticism of T&CM efficacy and safety, unregulated nature of T&CM practice, differences in the healing philosophies, HCPs’ perception that biomedicine is superior over T&CM and lack of knowledge about T&CM may explain HCPs’ unwillingness to refer Ebola survivors to T&CM practitioners [37, 51]. Although it was not reported in our study, the absence of a specific policy guideline on referral might limits collaboration between HCP and T&CM practitioners [28, 38]. Even though the traditional medicine policy in Sierra Leone advocates for the integration of the two main healthcare domains [33], the current picture suggests that conventional medicine and T&CM healthcare systems exist parallel to each other. Conventional medicine is the official medical model of healthcare, given that it receives government support and funding as well as developed infrastructure and human resource. Consistent with previous research findings in Africa [28, 29], our finding that all HCPs desired that T&CM practitioners should be properly educated, trained and regulated suggests that T&CM needs to be subjected to robust scientific and professional scrutiny as seen in the case of conventional medicine. This will help increase HCPs’ confidence in recommending the use of certain T&CM practices and referring Ebola survivors to T&CM practitioners. However, T&CM practice in Sierra Leone remains largely unregulated [52]. Also, rigorous research into the effectiveness and safety of commonly used T&CM in Sierra Leone is currently lacking [7, 52]. Thus, a clear policy and implementation strategy are needed especially in the areas of T&CM product research, T&CM practitioner education, training, and regulation as well as collaboration between the two disciplines to achieve the goal of integrating T&CM into the healthcare system in Sierra Leone. Specifically, these policy initiatives should focus on the establishment of T&CM research centres that promote the research and development of commonly used T&CM products and practices. Also, the creation of T&CM diploma and degree programs to formally train T&CM practitioners. In addition, an effective T&CM regulatory body needs to be established to ensure that T&CM products and practice meets ascribed quality assurance standards.

### Study limitation

Readers should consider the following limitations when interpreting our findings. Even though we interviewed HCPs in all four geographical regions of Sierra Leone, our finding cannot be generalised given the explorative qualitative designed we employed in our study. Also, HCPs from private healthcare facilities were not part of our study. Their views regarding T&CM may be different and, therefore warrant further research. The first author had a prior working relationship with three of the healthcare providers that participated in the study, and that might have affected their response that they provided. The views of healthcare providers reported in this study were in respect to Ebola survivors. However, given that the care received by Ebola survivors are part of the broader routine care that patients who are not Ebola survivors receive, healthcare providers’ views about T&CM are likely applicable to general patients.

## Conclusion

Our study suggests that HCPs’ negative perception of, and low level of knowledge about T&CM influenced their personal and professional use of T&CM, effective communication with Ebola survivors regarding T&CM as well as T&CM integration into the wider healthcare system in Sierra Leone. Educational interventions for HCPs such as workshops, distribution of evidence-based factsheets, the conduction of rigorous T&CM research, proper education and training of T&CM practitioners and effective professional regulation of T&CM practice could change the negative perception of, and increase evidenced base knowledge about T&CM among HCPs.

## Supplementary information

**Additional file 1.** Supplementary File 1. Consolidated Criteria for Reporting Qualitative Research (COREQ): 32-item checklist.

## Data Availability

The data used to support the findings of this study have not been made available because of the sensitive nature of its content and concerns surrounding privacy and confidentiality of research participants. Also, the University of Technology Sydney Human Research Ethics Committee and the Sierra Leone Ethics and Scientific Review Committee did not approve to share the raw data publicly. In addition, healthcare providers did not give consent for their data to be publicly shared. However, the anonymised raw data relevant to the study can be shared upon reasonable request on a case-by-case basis by contacting the following persons: Racheal Laugery, Senior research Ethics officer, University of Technology Sydney Human Research Ethics Committee, University of Technology Sydney, Email: (Racheal.Laugery@uts.edu.au);. Edward Foday, Research and Publications Specialist, Sierra Leone Ethics and Scientific Review Committee, Directorate of Policy, Planning and Information, Ministry of Health and Sanitation, Fifth Floor, Youyi Building, East Wing, Freetown, Sierra Leone, Email: efoday@health.gov.sl.

## Abbreviations

T&CM: Traditional and Complementary Medicine
TM: Traditional Medicine
HCP: Healthcare Providers
