# Supplementary material for: Traditional and complementary medicine use among Ebola survivors in Sierra Leone: a qualitative exploratory study of the perspectives of healthcare workers providing care to Ebola survivors
Source: BMC Complement Med Ther. 2020 May 6;20:137. doi: 10.1186/s12906-020-02931-6 (PMC7201764; doi:10.1186/s12906-020-02931-6)
Supplement: Supplementary file 1 — Additional file 1. Supplementary File 1. Consolidated Criteria for Reporting Qualitative Research (COREQ): 32-item checklist. [file 12906_2020_2931_MOESM1_ESM.docx]

**Supplementary File 1. Consolidated Criteria for Reporting Qualitative Research (COREQ):**

**32-item checklist**

| **Topic and Item No.** | **Guide Questions/Description** | **Reported on Page No.** |
| --- | --- | --- |
|  |  |  |
| **Domain 1: research team and reflexivity** |  |  |
|  |  |  |
| Personal Characteristics |  |  |
| 1. Interviewer/facilitator | Which author/s conducted the interview or focus | First author (Peter Bai James ) |
|  | group? | The first author conducted face-to-face semi-structured interviews in English  Page 7 |
|  |  |  |
| 2. Credentials | What were the researcher’s credentials? E.g. PhD, | Peter Bai James B.Pharm Hon, MMed  Jon Wardle PhD  Amie Steel PhD  Jon Adams PhD  Abdulai Jawo Bah B.Pharm Hon, MMed  Stephen Sevalie MD, MPH, MSc |
|  | MD |  |
|  |  |  |
|  |  |  |
|  |  |  |
|  |  |  |
|  |  |  |
| 3. Occupation | What was their occupation at the time of the | Pharmacist, PhD scholar, Research Assistant  Page 6 |
|  | study? |  |
|  |  |  |
| 4. Gender | Was the researcher male or female? | Male |
| 5. Experience and training | What experience or training did the researcher | He is trained in qualitative research and had experience in conducting semi-structured interviews among health professionals and patients in Sierra Leone. |
|  | have? |  |
|  |  | Page 6 |
|  |  |  |
|  |  |  |
|  |  |  |
| Relationship with participants |  |  |
| 6. Relationship established | Was a relationship established prior to study | PBJ worked with some of the participants prior to conducting this study.  Page 6 &20 |
|  | commencement? |  |
|  |  |  |
|  |  |  |
|  |  |  |
|  |  |  |
| 7. Participant knowledge of the | What did the participants know about the | Before the start of the interview, HCPs were given a participants information sheet that explains the nature and scope of the study as well as the researchers’ interest in the research topic.  Pages 7&8 |
| interviewer | researcher? *e.g. personal goals, reasons for doing* |  |
|  | *the* |  |
|  | *research* |  |
|  |  |  |
| 8. Interviewer characteristics | What characteristics were reported about the | Participants knew the researcher was a |
|  | interviewer/facilitator? e.g. *Bias, assumptions,* | Pharmacist and PhD scholar in Public health with an |
|  | *reasons and interests in the research topic* | interest in how Ebola survivors use informal healthcare approaches  Page 8 |
|  |  |  |
| **Domain 2: study design** |  |  |
|  |  |  |
| Theoretical framework |  |  |
| 9. Methodological orientation and | What methodological orientation was stated to | We used a qualitative content analysis exploratory study design using a semi-structured interview guide to collect our data.  Page 6 |
| Theory | underpin the study? *e.g. grounded theory,* |  |
|  | *discourse analysis, ethnography, phenomenology,* |  |
|  | *content analysis* |  |
|  |  |  |
| Participant selection |  |  |
| 10. Sampling | How were participants selected? *e.g. purposive,* | Snowball sampling |
|  | *convenience, consecutive, snowball* | Page 6 |
|  |  |  |
| 11. Method of approach | How were participants approached? *e.g. face-to-* | Telephone  Page 6 |
|  | *face, telephone, mail, email* |  |
|  |  |  |
| 12. Sample size | How many participants were in the study? | The final sample (n=15) was established when we reached data saturation during data collection. Page 6 |
| 13. Non-participation | How many people refused to participate or | None  N/A |
|  | dropped out? Reasons? |  |
|  |  |  |

| Setting | |  |  |
| --- | --- | --- | --- |
| 14. | Setting of data collection | Where was the data collected? *e.g. home, clinic,* | The interviews were conducted in their offices or consulting room after working hours, and it lasted between 30 and 95 minutes and were digitally recorded  Page 7 |
|  |  | *workplace* |  |
|  |  |  |  |
| 15. | Presence of non-participants | Was anyone else present besides the participants | No |
|  |  | and researchers? |  |
|  |  |  |  |
| 16. | Description of sample | What are the important characteristics of the | Age, sex, cadre, region and practice setting  See table 1 |
|  |  | sample? *e.g. demographic data, date* |  |
|  |  |  |  |
|  | |  |  |
| Data collection | |  |  |
| 17. | Interview guide | Were questions, prompts, guides provided by the | Yes, participants were asked to reflect |
|  |  | authors? Was it pilot tested? | on their interaction with Ebola survivors |
|  |  |  | The guide was pretested among three HCPs, and their response helped to inform the design of the final version of the interview guide  Page 6 |
|  |  |  |  |
|  |  |  |  |
|  |  |  |  |
| 18. | Repeat interviews | Were repeat interviews carried out? If yes, how | We did not conduct any repeat interviews, given that all the relevant areas were discussed in all of the 15 interview sessions.  Page 7 |
|  |  | many? |  |
|  |  |  |  |
| 19. | Audio/visual recording | Did the research use audio or visual recording to | Data were audio recorded using a |
|  |  | collect the data? | digital recorder  Page 7 |
|  |  |  |  |
| 20. | Field notes | Were field notes made during and/or after the | Yes  Page 7 |
|  |  | interview or focus group? |  |
|  | |  |  |
| 21. Duration | | What was the duration of the interviews or focus | Ranged between 30-95 minutes  Page 7 |
|  |  | group? |  |
|  |  |  |  |
| 22. | Data saturation | Was data saturation discussed? | Yes  page 6 |
| 23. | Transcripts returned | Were transcripts returned to participants for | Yes. The transcripts were sent via email to all participants to get their feedback on the accuracy of their response to the issues discussed during the interview. However, only six were able to respond  Page 7 |
|  |  | comment and/or correction? |  |
|  | |  |  |
| **Domain 3: analysis and findings** | |  |  |
|  | |  |  |
| Data analysis | |  |  |
| 24. Number of data coders | | How many data coders coded the data? | 4 |
| 25. | Description of the coding tree | Did authors provide a description of the coding | No |
|  |  | tree? |  |
|  | |  |  |
| 26. Derivation of themes | | Were themes identified in advance or derived | Themes were derived from the data  Page 8 |
|  |  | from the data? |  |
|  |  |  |  |
| 27. | Software | What software, if applicable, was used to manage | Data were transcribed verbatim into |
|  |  | the data? | Word documents by PBJ, and NVivo Software was used to manage and analyse our data  Page 8 |
|  |  |  |  |
|  |  |  |  |
|  |  |  |  |
| 28. | Participant checking | Did participants provide feedback on the findings? | No |
| Reporting | |  |  |
| 29. | Quotations presented | Were participant quotations presented to | Yes  Pages 8-16 |
|  |  | illustrate the themes / findings? Was each |  |
|  |  | quotation identified? *e.g. participant number* |  |
|  |  |  |  |
| 30. | Data and findings consistent | Was there consistency between the data | Yes |
|  |  | presented and the findings? | Pages 8-16 |
|  |  |  |  |
| 31. | Clarity of major themes | Were major themes clearly presented in the | Yes  Pages 8-16 |
|  |  | findings? |  |
|  |  |  |  |
| 32. | Clarity of minor themes | Is there a description of diverse cases or discussion | Yes  Pages 8-16 |
|  |  | of minor themes? |  |
|  |  |  |  |
